# Hypercapnea and Acidemia despite Hyperventilation following Endotracheal Intubation in a Case of Unknown Severe Salicylate Poisoning

**DOI:** 10.1155/2017/6835471

**Published:** 2017-03-29

**Authors:** Shannon M. Fernando, Valérie Charbonneau, Hans Rosenberg

**Affiliations:** ^1^Department of Emergency Medicine, University of Ottawa, Ottawa, ON, Canada; ^2^Division of Critical Care, Department of Medicine, University of Ottawa, Ottawa, ON, Canada

## Abstract

Salicylates are common substances for deliberate self-harm. Acute salicylate toxicity is classically associated with an initial respiratory alkalosis, followed by an anion gap metabolic acidosis. The respiratory alkalosis is achieved through hyperventilation, driven by direct stimulation on the respiratory centers in the medulla and considered as a compensatory mechanism to avoid acidemia. However, in later stages of severe salicylate toxicity, patients become increasingly obtunded, with subsequent loss of airway reflexes, and therefore intubation may be necessary. Mechanical ventilation has been recommended against in acute salicylate poisoning, as it is believed to take away the compensatory hyperpnea and tachypnea. Despite the intuitive physiological basis for this recommendation, there is a paucity of evidence to support it. We describe a case of a 59-year-old male presenting with decreased level of consciousness and no known history of ingestion. He was intubated and experienced profound hypercarbia and acidemia despite mechanical ventilation with high minute ventilation and tidal volumes. This case illustrates the deleterious effects of intubation in severe salicylate toxicity.

## 1. Introduction

Severe, acute salicylate toxicity remains a common presentation to the Emergency Department (ED) and is associated with a significant degree of mortality [1]. In its unionized form, salicylate can move across cell membranes into tissues to exert toxic effects. In the presence of acidemia, salicylate will shift to this unionized form, which allows it to cross the blood-brain barrier, and cause central nervous system toxicity (cerebral edema, seizures, and coma). Therefore, the presence of acidemia is seen as a poor prognostic indicator. Classically, salicylate toxicity is initially associated with a respiratory alkalosis, secondary to direct stimulation of the medulla, and subsequent tachypnea and hyperpnea as a response to metabolic acidosis. For this reason, intubation and mechanical ventilation have been commonly recommended against in severe salicylate poisoning, as it is believed that this intervention may take away this protective respiratory drive [2, 3]. Other sources suggest that intubation may be safely performed, as long as apneic time during induction is minimized, and the patient is hyperventilated adequately on the ventilator [3]. Unfortunately, very little evidence exists on this topic, and there are multiple reasons that a patient with salicylate toxicity may require intubation, including decreased or altered level of consciousness, failure to protect airway, and respiratory distress from pulmonary edema. Taken together, there is very little understanding regarding the approach to intubation of patients with severe salicylate overdose. We present a case of a patient with unknown severe salicylate toxicity, who was intubated upon arrival, but immediately hyperventilated several minutes later once initial blood gas levels revealed the diagnosis. To our knowledge, we are the first to publish pre- and postintubation blood gas data in the context of ventilator settings that should have resulted in hyperventilation and improving acidemia.

## 2. Case Report

The patient was a 59-year-old male farmer who had been found by his daughter to be confused and tachypneic on the morning of presentation, and she brought him to the local community ED. His vital signs were blood pressure of 116/75 mmHg, heart rate (HR) of 107 beats/minute, respiratory rate (RR) of 20 breaths/minute, initial temperature of 36.3 degrees' Celsius, and 100% oxygen saturation. Glasgow Coma Scale (GCS) was 15 on initial physician assessment. His weight was estimated at 65 kg. He was treated for presumed pneumonia with moxifloxacin and transferred to our tertiary care hospital for computed tomography (CT) scan of the head, in order to rule out intracranial pathology. During transfer, paramedics assessed the patient and found him to be significantly obtunded, with a GCS of 7.

Upon arrival at our ED, the patient had a HR of 130 beats/min, a RR of 15–20 breaths/min, and a stable blood pressure. He had a temperature of 39 degrees' Celsius and was diaphoretic and mottled to the chest. GCS remained at 7. Pupils were equal and reactive and the physical exam was otherwise unremarkable. Bedside glucose was obtained and was 5.3 mmol/L. A venous blood gas (VBG) and standard sepsis bloodwork (including blood cultures) were sent upon presentation. At this point, there was concern regarding the rapid decline in level of consciousness without a clear etiology. It was decided that an urgent CT scan of the head was indicated and since the patient would not obey commands and was not lying still, the decision was made to intubate him in order to facilitate the scan. The patient was induced for rapid sequence intubation (RSI) with propofol (50 mg) and rocuronium (50 mg). Following administration of propofol, the patient was ventilated using bag mask ventilation, in order to facilitate gas exchange during induction. He was successfully intubated using a GlideScope® on the first attempt. Nursing notes suggest that the longest possible apnea time was approximately 4 minutes, which reflects the time from rocuronium administration to detection of end-tidal carbon dioxide. A postintubation chest X-ray revealed a possible small consolidation in the left lower lung, but no evidence of pulmonary edema to suggest acute respiratory distress syndrome (ARDS).

Several minutes following intubation, the patient's preintubation bloodwork returned. VBG revealed a pH of 7.37, pCO2 of 19, HCO_3_ of 11, and venous base excess of −11.1 mmol/L. This mixed respiratory alkalosis and metabolic acidosis were suggestive for salicylate poisoning, and ventilator settings were adjusted to increase the respiratory rate (increased to 25, as compared to the patient's initial 20 breaths/minute) and tidal volume (increased to 620 mL, approximately 10 mL/kg of total body weight, as ideal body weight was unknown), which were the maximal tolerated settings. At this time, a toxicologic work-up was added to the bloodwork, and collateral history of ingestion was obtained, revealing access to 240 mg acetylsalicylic acid (ASA) boluses used for cattle. It was later confirmed that the patient had in fact intentionally overdosed on this ASA, though his family was not aware of this.

The patient was given two bolus ampules of IV NaHCO_3_ and started on a NaHCO_3_ infusion. The serum salicylate level was found to be critically elevated at 7.2 mmol/L. VBG performed twenty minutes following intubation revealed a pH of 6.89, pCO2 of 121, HCO_3_ of 23, and venous base excess of −13.2 mmol/L. A 50 g/L dextrose infusion was started to counter possible neuroglycopenia (which was presumed to be contributing to the decreased level of consciousness), and Nephrology and Critical Care were consulted for urgent hemodialysis. Despite the above interventions, a repeat VBG twenty minutes after the previous showed a pH of 7.03 and pCO2 >120 (HCO_3_ and venous base excess were not reported). Soon after, the patient suddenly became very bradycardic and arrested. Initial rhythm was consistent with pulseless electrical activity (PEA). Chest compressions were started and the patient received bolus doses of 1 mg epinephrine and 50 mEq NaHCO_3_. Return of spontaneous circulation was briefly achieved, but subsequently lost again several minutes later. The patient again received multiple doses of epinephrine, NaHCO_3_, and 1 g calcium chloride. Bedside ultrasound confirmed absence of cardiac activity. The resuscitation was terminated after thirty minutes, at which point the patient's family requested to cease resuscitative efforts. CT of the head was ultimately not completed. The remainder of the toxicologic work-up (acetaminophen, ethanol, ECG) did not reveal any obvious coingestion.

## 3. Discussion

Acute salicylate poisoning remains an important consideration in the undifferentiated patient with altered mental status, due to its subtle signs and significant mortality. While intubation of these patients may sometimes be necessary, it is commonly recommended against by several sources, due to the belief that it may stifle respiratory compensation and worsen acidemia. That being said, there are no experimental trials to support this belief, and only a few case reports exist (with varying results). In our literature review, we found one case report supporting the notion of worsening acidemia with intubation in severe salicylate overdose [4]. However, we also noted a case report of successful intubation in a patient with an intentional ASA overdose, though the serum concentration of salicylate was unknown [5]. The most comprehensive literature on this topic was a case-series published by the New York City Poison Control Centre, which was a retrospective review of 3,144 cases of salicylate poisoning from 2001 to 2007 [6]. In this 8-year time period, these authors found only 7 cases with available blood gas data of intubation in severe salicylate poisoning. In all of these cases, intubation resulted in acidemia, though the preintubation pH was not always known. Two of these 7 cases resulted in death. Furthermore, full blood gas values are not provided. Therefore, it is unclear how much of this acidemia is secondary to metabolic acidosis from salicylate metabolism and how much is secondary to respiratory acidosis from intubation and reduced respiratory drive. Finally, the time periods at which these pH values were collected are unknown, and evidence demonstrates that peak concentration of serum salicylate in acute overdose may not reach peak values until 6 hours following ingestion, due to bezoar formation [7, 8]. In our case, acidemia can be attributed to both respiratory acidosis (evidenced by hypercarbia) and likely metabolic acidosis from salicylate metabolism.

Several sources on management of acute salicylate toxicity also recommend that, if proceeding with intubation, the clinician should try to minimize the time of apnea and try to match the patient's minute ventilation with the ventilator [3, 6]. However, again there is no data available regarding either of these factors in intubation of these patients. In our case, the time from induction to airway security was less than four minutes, which should have minimized time of apnea. Further to that, the ventilator settings were adjusted in an attempt to maintain the patient's respiratory alkalosis. The patient's initial respiratory rate was 20, and this was increased to 25 on the ventilator. His tidal volume was unknown but was set at 620 mL (roughly 10 mL/kg of total body weight, as ideal body weight was unknown). There is no data available regarding the degree of hyperpnea seen in salicylate overdose. Evidence seems to indicate that normal tidal volume is roughly 6–8 mL/kg of ideal body weight [9], suggesting that this patient received tidal volumes and minute ventilation that should be consistent with hyperventilation. However, despite this, the patient continued to have worsening hypercapnia. This may support suggestions that mechanical ventilation is simply incapable of maintaining the necessary ventilation to prevent acidemia. It is also worth noting that our patient was paralyzed with rocuronium, which should have improved hyperventilatory effort from mechanical ventilation.

Uniquely, we are the first to demonstrate worsening acidemia despite ventilator settings that should have matched the patient's hyperventilation, lending credence to the notion that mechanical ventilation may be unable to provide the respiratory support required to maintain respiratory alkalosis in these patients. Ultimately, this case supports the notion that intubation should remain as a very last resort in the setting of acute salicylate toxicity.

## 4. Conclusion

Severe salicylate toxicity is a life-threatening condition, and in the absence of an obvious history of ingestion, the diagnosis can only be made with a high degree of suspicion. Furthermore, the sequelae of toxicity on multiple organ systems suggest that the initial presentation can be quite varied. In any case of undifferentiated altered mental status, the instinct of the Emergency Physician is to first focus on the patient's ability to maintain their airway. This often results in endotracheal intubation. However, in the case of severe salicylate toxicity, intubation can result in worsening acidemia and cardiac arrest. Sometimes the only clue to the diagnosis is tachypnea and hyperpnea, and therefore all clinicians should be wary of the patient that appears to have rapid minute ventilation, as this patient may be attempting to maintain their neutral pH in the face of mounting acidosis. Further to this, should there be no choice but to intubate a patient in this condition, the clinician must do their best to maintain the large minute ventilation. However, as demonstrated in our case, that may not be feasible through mechanical ventilation.
